# Microwave-Assisted Synthesis as a Promising Tool for the Preparation of Materials Containing Defective Carbon Nanostructures: Implications on Properties and Applications

**DOI:** 10.3390/ma16196549

**Published:** 2023-10-04

**Authors:** Damian Pawelski, Marta E. Plonska-Brzezinska

**Affiliations:** Department of Organic Chemistry, Faculty of Pharmacy with the Division of Laboratory Medicine, Medical University of Bialystok, Mickiewicza 2A, 15-222 Bialystok, Poland; damian.pawelski@sd.umb.edu.pl

**Keywords:** carbon nanostructure, defect, heteroatom doping, catalysis, electrocatalysis, electrochemistry, supercapacitor, microwave irradiation, microwave-assisted synthesis, inorganic nanoparticle

## Abstract

In this review, we focus on a small section of the literature that deals with the materials containing pristine defective carbon nanostructures (CNs) and those incorporated into the larger systems containing carbon atoms, heteroatoms, and inorganic components.. Briefly, we discuss only those topics that focus on structural defects related to introducing perturbation into the surface topology of the ideal lattice structure. The disorder in the crystal structure may vary in character, size, and location, which significantly modifies the physical and chemical properties of CNs or their hybrid combination. We focus mainly on the method using microwave (MW) irradiation, which is a powerful tool for synthesizing and modifying carbon-based solid materials due to its simplicity, the possibility of conducting the reaction in solvents and solid phases, and the presence of components of different chemical natures. Herein, we will emphasize the advantages of synthesis using MW-assisted heating and indicate the influence of the structure of the obtained materials on their physical and chemical properties. It is the first review paper that comprehensively summarizes research in the context of using MW-assisted heating to modify the structure of CNs, paying attention to its remarkable universality and simplicity. In the final part, we emphasize the role of MW-assisted heating in creating defects in CNs and the implications in designing their properties and applications. The presented review is a valuable source summarizing the achievements of scientists in this area of research.

## 1. Introduction

The term “nanoscale” is applied to objects up to 100 nm in size. It also applies to structures built of many atoms, forming groups called nanostructures because their size falls within this scale. These consist mainly of carbon atoms (carbon nanostructures, CNs), whose combination in well-organized macromolecular systems creates structures with unique properties, and they are among the most popular in this group [1,2,3]. Different morphological variations of CNs, for example, nanocones [4], nanotubes [5], diamond-like carbon [6,7], onion-like carbon [8], nanofibers [4], graphene [9], and graphdiyne [10,11,12], have become quite well known [4,8,9,13,14]. Despite almost identical chemical compositions, the CNs differ significantly in their physical properties with different dimensionality and chemical reactivity [3,15]. These greatly affect their subsequent use in many areas, mainly in electronics [16,17], catalysis [18,19], electrocatalysis [20], energy conversion and storage [21,22,23], sensors [24,25,26,27,28], and for biomedical [29], biological, and environmental applications [30,31,32,33]. The electrochemical and electrocatalytic properties of carbon-based materials are closely related to their chemical composition and structure [34,35,36]. CNs are characterized by excellent conductivity and mechanical stability, large specific areas (active surface) with micro- and mesoporosity, and high mobility of the charged carrier [37,38,39,40], which only strengthens their position in the abovementioned areas. CNs can be easily modified by producing numerous defective motifs, shortly called defects, with different chemical characters that enhance the physicochemical properties of the materials. Defects in nanomaterials are considered ‘active sites’ of the reaction because they change the electron density in the material, affecting the electronic and surface properties in the local region [41,42,43]. As for photocatalysis [44,45] and electrocatalysis [46,47,48], reactions occur on the contact interface between ‘active sites’ of materials and reactive species. The defects modify the electronic structure of the CNs, simultaneously affecting the chemisorption of the key reaction intermediates [41]. This process enhances the reaction kinetics, consequently increasing electrocatalytic efficiency [49,50]. In terms of energy-storage devices, carbon-based materials are generally used as electrodes in electrochemical capacitors [35,51,52]. The energy storage mechanism in supercapacitors (SCs) is based on reversible ion adsorption on a high specific surface area (SSA) of porous carbon materials at the carbon/electrolyte interface [53]. It was also found that defects in sp^2^-hybridized CNs significantly improved ion storage performance [54]. Thus, the contact area at the interface is crucial for efficient electrocatalytic reactions and electrochemical storage devices.

Structural defects can be of various natures and can be introduced into the “ordered” structure during the formation of CNs or as a result of post-modification processes. Direct methods for producing defective CNs (d-CNs) include chemical vapor deposition (CVD) [55,56,57], thermal treatment [58,59], pyrolysis [60,61], microwave (MW) irradiation [62,63,64,65], etc. Using different process parameters and substrates, it is possible to produce pristine d-CNs or more complex systems containing, for example, heteroatomic dopants or inorganic components [66,67,68,69]. A post-modification process is often also used, which leads to introducing external defects into the CN’s surface. It is used as one stage of the carbon-based materials production processes or to modify the already formed CNs. The latter case is frequently connected with chemical treatment, which uncontrollably introduces defects in the outer layers of CNs with the simultaneous creation of functional groups on the nanostructure’s surface due to the redox reaction of the C atoms of the outer carbon layer [48,70,71,72,73]. CNs can be modified in more controllable manner using, for example, plasma-induced functionalization [74,75] or MW irradiation [76,77]. In these cases, the concentration of topological defects introducing functional groups or heteroatom dopants can be controlled by the appropriate selection of process parameters.

All these aspects will be discussed in more detail in our review. We decided to discuss the literature related to MW-assisted synthesis used in the creation of d-CNs due to its simplicity, its economy, and the possibility of conducting the reaction in solvents and solid phases in the presence of components of different chemical natures, which allows the use of them in various combinations. It is the first literature review that deals with the use of MW-assisted heating to modify the structure of CNs, which are aimed at introducing topological changes that, consequently, modify their physical and chemical properties and, therefore, decide on their further application. This review focuses on the materials containing pristine d-CNs and those incorporated in the hybrid materials. We will emphasize the advantages of synthesis using MW-assisted heating and the implications for the chemical and physical properties of CNs. In the final part of the work, we present examples of the use of MW-assisted heating to obtain materials that have promising properties and can be used in electrochemistry and electrocatalysis.

## 2. Defects and Their Structural Variation

Defects can be divided into four categories based on their size and location [41,78,79,80]:

1. Zero-dimensional (0D) point defects (e.g., doping, vacancy, reconstruction), which can be further divided into reconstructed or vacancy defects, non-metallic-atom-doping induced defects, and metal defects [42,81].
2. One-dimensional (1D) line defects (e.g., dislocation).
3. Two-dimensional (2D) planar defects (e.g., grain boundary).
4. Three-dimensional (3D) volume defects (e.g., spatial lattice disorder).

The disorder in the crystal structure is related to the ideal lattice structure [82,83]. In 2D materials, such as graphene, the types of defects are very diverse [84]. Point defects are on a tiny scale, referring even to the defect of a single atom in the crystal. Due to the location of this defect, we distinguish among them vacancy, dislocation, interstitial defect, replacement, and antisite. Line defects are related to disturbances in the crystal structure along the crystalline (for example, grain boundaries). Surface defects have a very diverse nature. It is defined as any disturbance of the crystal structure in one direction. Here, we can indicate, for example, twin crystals, grain boundary, hole, etc. The last group of defects, the largest in their scale, are defects related to 3D structure.

In electrochemistry, where reactions occur on the electrode surface, the electrode materials can be considered basal and non-basal surfaces. Carbonaceous materials, including graphene and graphite derivatives, are used as electrode materials, with ideal model structures for studying electrochemical processes. We can distinguish basal and non-basal surfaces in both systems, including edge surfaces and defect surfaces [85]. The ideal basal surfaces are considered a well-organized layer of only six-ring carbons. The presence of pentagonal and heptagonal rings, pentagon–heptagon pairs, and occasional quadrangular and octagonal ones in the graphene layer is defined as in-plane topological defects (non-basal surfaces) [86]. 

**Figure 1:**
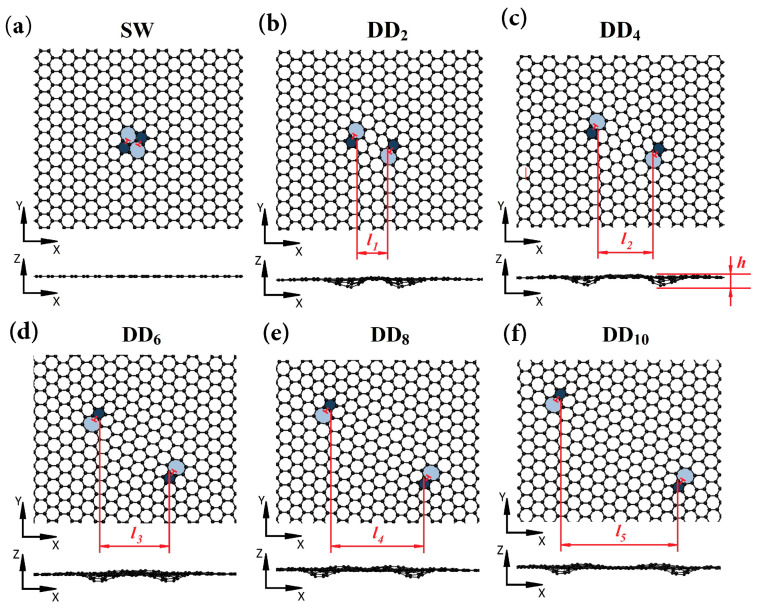
Typical structures of graphene in two projections: (**a**) graphene with SW defect, (**b**–**f**) graphene with dislocation dipole (DD_𝑛_) with the different arm, the subscript *n* indicates the distance between two 5–7 pairs in the hexagon units. Pentagons and heptagons are colored in dark and light blue, respectively. Geometrical parameters for dipoles, values of dipole arm (*l*) and buckling height (*h*) for graphene with the following defects: SW (*l* = 0; *h* = 0); DD_2_ (*l* = 6 Å; *h* = 1.89 Å); DD_4_ (*l* = 11 Å; *h* = 2.0 Å); DD_6_ (*l* = 15 Å; *h* = 1.25 Å); DD_8_ (*l* = 20 Å; *h* = 1.22 Å); DD_10_ (*l* = 25 Å; *h* = 1.78 Å) Reprinted with the permission from MDPI, Ref. [88].

The combination of tightly bounded pentagons and neighboring heptagons (Stone–Wales (SW) defect) can be identified as an edge dislocation in a 2D hexagonal lattice (Figure 1a) [87]. The SW defects are created by rotating a C–C bond, transforming the four adjacent hexagons into two pentagons and two heptagons [88]. If the SW defect is separated through hexagonal units into two separate pentagon–heptagon connections, dislocation dipoles (DD_n_) appear in the graphene structure. The subscript *n* indicates the distance between two 5–7 pairs in the hexagon units (distance *l*, Figure 1b–f). DDs are not only in-plane surface defects but also cause out-of-plane ring deviation due to introducing irregular defects into the graphene layer (height *h*, Figure 1c–f).

The differentiation of defects is also introduced, considering materials’ physical and chemical properties. Here, we can distinguish Frenkel defects (interstitial atoms) [89], Schottky defects (carbon vacancies) [89], heteroatom–doping (doping defects) [66], electronic defects in the crystal [90], and nonstoichiometric structured defects [91], etc. No matter how the structural defects in CNs are subdivided, they refer to some disturbance of the structure compared to the ‘ideal structure’ of the nanomaterials. This ‘disturbance’ changes some specific features of the nascent nanostructure, consequently modifying the physical and chemical properties of the CNs.

## 3. Heteroatom Doping and Structural Defects of CNs with Relevance in Electrocatalysis and Electrochemistry

Carbon materials act as a catalyst or materials with satisfactory electrochemical or electrocatalytic performance. However, amorphous or disordered carbon materials exhibit low activity, chemical, thermal and electrochemical stability, and low oxidation resistance [92,93,94]. The combination of carbon atoms in a 2D or 3D manner increases its stability, and at the same time, an organized macromolecular architecture defines its physical and chemical properties.

Carbon materials used as electrodes in capacitors show a direct relationship between the electrostatic capacitance and their SSA values [95]. However, nanoscale systems do not directly comply with the above rule. In this case, the nanoscale and quantum effects must be frequently considered because it has a large surface–to–volume ratio. In this case, we observe an increased role of the material’s surface in the capacitive storage process [96]. It is essential to not only the total number of pores but also their size and arrangement of pores. Carbon defects of various natures, such as dislocations, atomic vacancies, and stacking faults, comprise the surface active regions (ASA) in catalytic/electrocatalytic reactions [97] or SSA in electrochemistry. The whole is a structural feature of graphitic CNs [98]. Several experimental and theoretical studies have shown that the experimental conditions during the creation of defects influence the CNs’ ASA and SSA [55,98].

**Figure 2:**
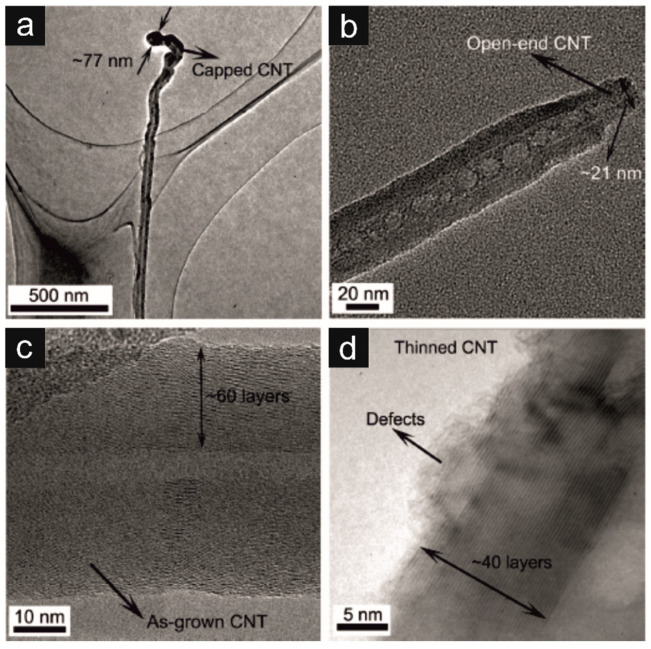
Low–resolution TEM images of (**a**) capped CNTs and (**b**) thinned and open-ended CNTs. HR–TEM images of (**c**) as–grown CNTs with 60 layers and (**d**) MW–assisted H_2_ plasma–processed CNT with ~40 layers and defected outer shells. Reprinted with the permission from Elsevier, Ref. [99].

Microstructural variations of CNs are also strongly connected with their conductivity and field emission. High–performance field emission (FE) requires low turn–on and threshold fields, good electrical and mechanical stability, and dense and uniform emission sites [99]. The carbon nanotubes (CNTs) with excellent conductivity meet the abovementioned criteria. In Figure 2b,d, on the transmission electron microscopic (TEM) images, the influence of the MW-assisted H_2_ plasma process on the structural changes of the CNs is presented. The thinned and open-ended caps of the CNTs can yield larger FE. Raman spectroscopy enabled a qualitative assessment of the created CNs by comparing the intensity of the starting material’s D– and G–bands (*I_D_*/*I_G_*) and those subjected to plasma treatment. Increasing the *I*_D_/*I*_G_ ratio with MW irradiation time indicates the formation of defects in the structure of CNTs. The created sp^3^-hybridized defects (defected graphite) on the CNT’s walls may act as new emission sites [100,101,102]. Plasma treatment also reduces the number of graphene layers and cup openings in CNTs (Figure 2b–d) [99]. Open–ended CNTs can facilitate electron tunneling and significantly improve the physical parameters of d-CNTs. The most significant is the high emission current density of 10.36 mA/cm^2^. In summary, MW irradiation and H_2_ plasma processing increase electron transferring traces and enhance CNT’s FE performance.

**Figure 3:**
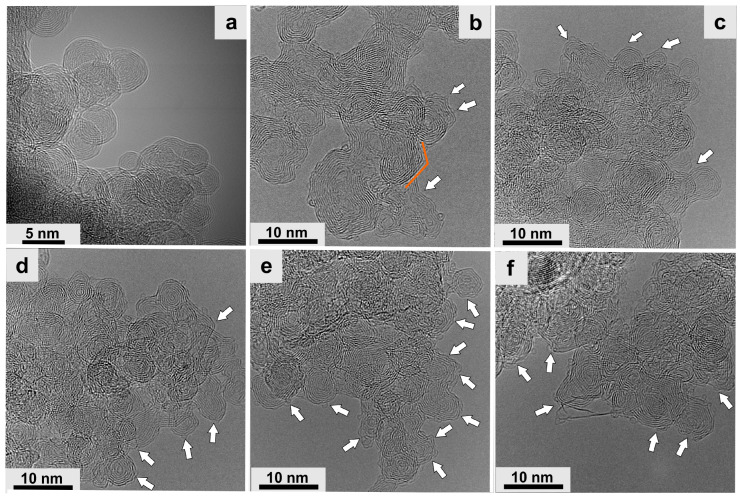
High–resolution TEM images of CNOs obtained from annealing of NDs at 1650 °C in He atmosphere (**a**) non–modified CNOs, (**b**) B–doped CNOs, (**c**) N–doped CNOs obtained from aminated–NDs, (**d**–**f**) and modified using MW heating in the presence of different oxidative agents (unpublished results of our group). White arrows indicate different types of structural defects. The orange line highlights the polygonal nature of the CN.

The studies confirmed that defects in graphitized carbons at the nanoscale significantly enhance the electrochemical behavior in aqueous electrolytes. For example, the use of ‘small’ spherical nanostructures (Figure 3a), called carbon nano–onions (CNOs) in energy storage devices, has enabled high power and high energy [103]. Comparing the CNO parameters to conventional carbon black and graphite nanoparticles in these devices allows for better gravimetric and volumetric parameters [1,103]. For example, CNOs obtained from nanodiamond (ND) particles at 1800 °C showed a high SSA, determined using the Brunauer–Emmett–Teller theory (*S_BET_*), of about 680 m^2^/g [104], lower than that of many carbon materials [105,106,107]. Despite this, the external surface is fully available for the adsorption of ions from the solution, which allows for achieving optimal parameters of devices accumulating an electric charge [108,109].

An important parameter known to influence electrocatalytic and electrochemical properties is surface chemistry [110,111,112,113]. So far, two main strategies have been used for the surface modifications. The first strategy involves the post–synthetic treatment of already formed CNs using various oxidizing or reducing agents [114,115,116,117]. The second is based on the transformation of the structure by introducing heteroatoms during the formation of CNs, which is called doping [118,119]. For example, doping CNOs with boron (B–CNOs) causes a decrease in the electrical conductivity value due to breaking the outer layer of CNOs [69]. ND-derived CNOs in thermal treatment are spherical structures, mainly with sp^2^–hybridized carbon atoms (Figure 3a). A small addition of pentagons has been identified in systems, like in single–layered fullerenes, which provide structure closure and surface curvature. These topological variations are sp^3^–hybridized and are called defects in sp^2^–hybridized CNs. In Figure 3, some defects are marked with white arrows for visibility. The results obtained by our group showed that the direct doping of CNO with B and N causes the graphitization of the structure and polygonization (Figure 3b). CNOs are in a compressed state, and the spacing between the CNO layers ranges from 0.32 nm to 0.27 nm compared with undoped CNOs (0.34 nm) [34]. The observed structural changes in CNs result in improved electrochemical and electrocatalytic properties of CNs due to numerous defects affecting the increase in porosity and, consequently, the specific surface [34,120].

In many cases, the methods of obtaining CNs at very high temperatures make it impossible to use organic reagents; therefore, to optimize the physical properties of CNs, it is necessary to use post-synthetic treatment. One of the simplest and most frequently used methods leading to numerous structure defects is the chemical oxidation of CNs using various oxidizing reagents [72,121,122,123,124]. There are several methods in the literature where oxidation with a mixture of acids predominates, e.g., HNO_3_ or a combination of HNO_3_ and H_2_SO_4_ [72,121], KMnO_4_ [122], HCl [123], H_2_O_2_ [124], or ozone [73]. Chemical treatment leads to the structural degradation of CNs, introducing numerous defects of various sizes, even those that lead to a complete break in the continuity of subsequent layers. In Figure 3d–f, high–resolution TEM images show CNOs subjected to chemical treatment using different oxidative agents. MW irradiation (300 W) was used to obtain CN surfaces with various defects for several minutes. The oxidation of CN surfaces may also be achieved by multiple methods, such as wet chemical oxidation [72], constant potential electrolysis [125], sonochemical or plasma treatment [123,126], etc. In parallel, C atoms are oxidized, forming functional groups containing oxygen, such as aldehydes, ketones, epoxides, esters, alcohols, and carboxylic acids [70,73]. Many works postulate that the presence of oxygen in CNs inhibits the electrocatalytic activity of nanostructures [127,128]. In contrast, oxygen–containing functionalities enhanced the electrochemical properties of CNs [70,129,130]. Additionally, numerous structure defects in the form of holes increase SSA, positively affecting carbon materials’ electrochemical properties.

**Figure 4:**
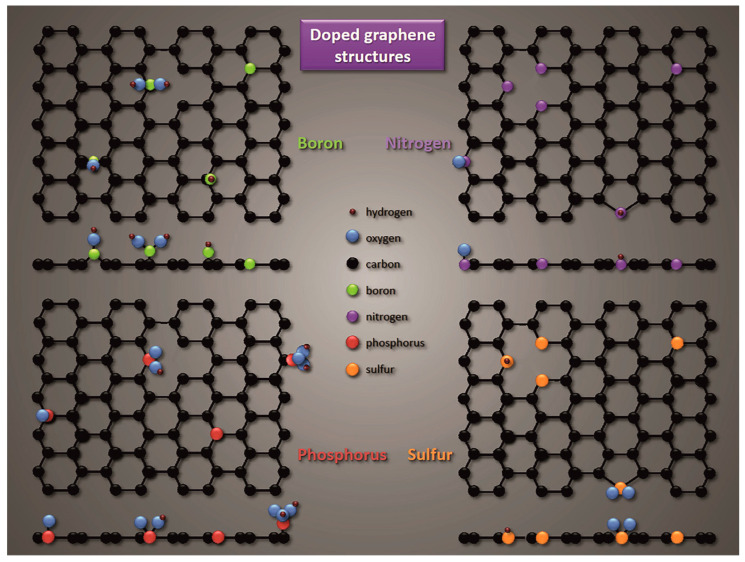
Schematic representation of possible positions for dopant atoms in the graphene network. In each case, the normal and cross–sectional views of the doped material are provided for easier visualization. The representation does not include the actual number of chemical bonds between the elements. Reprinted with the permission from Elsevier, Ref. [135].

The chemical functionalization of the CN surface also includes a doping that modulates the CN structure [120,131,132,133], creating defects and, at the same time, increasing the catalytic and electrocatalytic activity [48,117,120,134]. Introducing non–metal dopants into the carbon layer may lead to charge polarization between the heteroatom–doped and the adjacent C atoms due to their different electronegativity [49,135]. Among them, the following stands out (Figure 4) [135]:

i. N and O acting as electron acceptors or donors for the adjacent C [81,134,136,137];
ii. B, F, S, and P acting as electron donors for the adjacent C [138,139,140].

Some examples of heteroatom doping are presented in Table 1. The heteroatom incorporation could trigger electron transfer to enhance the electrical conductivity of CNs. On the other hand, the electrical conductivity is determined by the carrier concentration, specific band structure near the Fermi level, and charge distribution. As mentioned earlier, incorporating heteroatoms into the CN creates defects in the structure by changing the local charge and consequently affects the electrical conductivity of the entire nanostructure [55]. The decisive parameters are the charge concentration and distribution (the nature and amount of doped heteroatoms) and the specific structure of the band near the Fermi level [113]. In CNs, localized states appear in the electronic band structure, transforming the passive graphitic network into CNs enriched with reactive sites that interact more strongly with molecules from the external environment [141].

Doping is a critical process that significantly affects the conductive properties of carbon materials, creating electron holes (p–type doping) in the graphene layer or places with an excess negative charge (n–type doping) [118]. Because of the π–conjugation, sp^2^–hybridized CNs can be electron donors or acceptors when coupled with other elements with different electronegativity. Breaking the integrity of the π–conjugation leads to the release of carbon π electrons [37,138,140,142,143]. In N–doped CNs, coupling the C atoms with the lone pair of electrons of the N atoms occurs; as a result, the C atoms adjacent to the N become active [37,144]. Based on measurements made using X–ray photoelectron spectroscopy (XPS), three main groups of N dopants were distinguished (Figure 4):

1. Pyridinic–like N is an atom that combines with two C atoms, enriching the aromatic ring with one p electron (p–type).
2. Pyrrolic–like N is an atom that bonds to two C atoms, sharing two p electrons with the π system (n–type).
3. Graphitic–like N (quaternary) in the graphene layer (in the six–membered ring) is substituted for one C atom (n–type).

**Figure 5:**
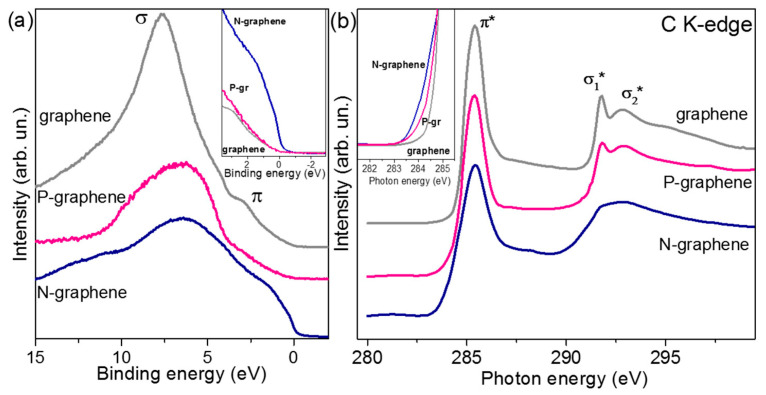
(**a**) Valence band XPS and (**b**) near–edge X–ray absorption fine structure (NEXAFS) C K-edge spectra of the annealed graphene, N–graphene, and P–graphene films. Insets show the spectra near the Fermi level. Reprinted with the permission from MDPI, Ref. [66].

We can determine the electronic properties of CNs using, for example, XPS or Near–Edge X–ray Absorption Fine Structure (NEXAFS) C–edge methods (Figure 5) [66]. XPS measurements should be performed for a skinny graphene film, which allows for the determination of changes in the energy states of the CN after its doping with heteroatoms (Figure 5a). The spectrum of the undoped graphene layer is analogous to that recorded for sp^2^–hybridized carbons [145]. The dominant peak in the spectrum of undoped graphene corresponds to the occupied σ states, and the π states of graphene form the peak at approximately 3.0 eV. The inset in Figure 5a compares spectra, ranging from −2.5 eV to 2.5 eV, normalized to the maximum intensity. In the range of positive values, undoped graphene and P–doped graphene have a similar density of occupied states, while doping with N atoms does not cause a significant increase in the electron density value in the valence band [146]. Unoccupied carbon states in graphene layers were investigated using the NEXAFS C–edge method (Figure 5b). The spectra of undoped graphene and N– or P–doped graphene illustrate electron transitions from the C 1s levels to the partially occupied and empty π and σ states [147]. These transitions use the π* resonance at 285.4 eV and the σ* resonance at 291.7 and 292.8 eV [147]. In the graphene spectrum, an apparent splitting of the σ* resonance into σ_1_* and σ_2_* is visible, indicating the high crystallinity of graphene layers [148]. A band smoothing in the σ* range is observed for N–doped graphene, indicating numerous defects in the N–doped CNs [149]. The inset in Figure 5b compares the low–energy region of the K–edge C spectra, where the density of unoccupied states increases in the series undoped graphene > P–doped graphene > N–doped graphene. It means that N-doped graphene has the most vacancies and edge defects. Therefore, the low conductivity of graphene layers doped with heteroatoms results from the scattering of charge carriers by heteroatoms and topological defects at the domain boundaries [150].

Additionally, heteroatom doping, which creates the pyridinic– and pyrrolic–like N atoms, frequently incorporates holes (topological defects) in the graphene layer (Figure 4), which may affect the optimization of the electrochemical properties of the nanostructure by facilitating electrolyte penetration and ion transport. It should also be noted that introducing the N atom into the graphene sheets causes a shift in the conduction band, allowing additional electrons to be adopted. A comparison of the electronic structures of N–doped graphene indicates that graphene with pyridinic–like N systems has the strongest electron deficiency for electron-accepting [81]. Therefore, the pyridinic–like defects are suitable as anode for Li batteries. It has also been shown that quaternary–like defects are suited for Li–ion batteries and offer a lower diffusion and desorption barrier than undoped graphene [139].

**Figure 6:**
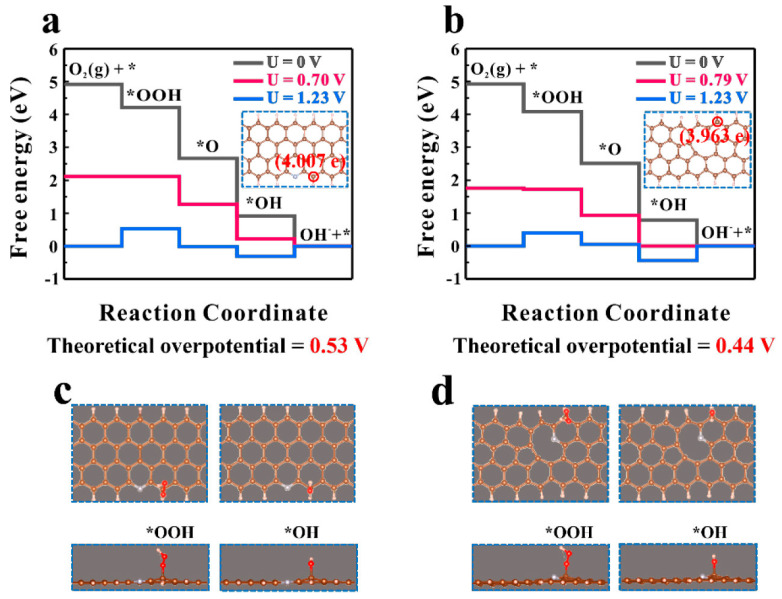
(**a**) Charge ORR free energy diagrams of (**a**) conventional pyridinic N site and (**b**) N–HPC site, optimized adsorption configuration of ORR intermediated (∗OOH and ∗OH) on (**c**) conventional pyridinic N and (**d**) N–HPC site (brown, silver, red, and incarnadine balls represent the C, N, O, H atoms, respectively). Reprinted with the permission from American Chemical Society, Ref. [151].

The pyridinic-like defects exhibit the nature of Lewis bases, which make them oxygen reduction reaction (ORR) active sites that catalyze O_2_ dissociation reactions (Figure 6 and Table 1) [151]. Additional topological defects can enhance the catalytic activity of pyridinic–like defects. It was demonstrated that monovacancy–coupled pyridinic N sites tuned the electronic properties of C-N bonds. Using the density functional theory (DFT), an energy difference was demonstrated between pyridinic N sites and monovacancy–coupled pyridinic N sites, 398.4 eV and 397.7 eV, respectively. This is related to the different charge distribution within the defects, −0.253 e (pyridinic N sites) and −0.225 e (monovacancy–coupled pyridinic N sites). It, in turn, causes stronger adsorption of oxygen–containing intermediates on the N–doped hierarchical porous carbon (N–HPC) surface and affects the ORR kinetics (Figure 6a–d). The kinetic current density (*i_k_*) for ORR is 19.11 mA/cm^2^ at 0.8 V in an alkaline solution (0.1 M KOH), which is higher than for Pt/C (18.06 mA/cm^2^) in the same experimental conditions. The N–HPC materials in alkaline solution exhibit an intrinsic turnover frequency of 7.26 times higher than typical pyridinic N atoms, and this is one of the highest values appearing in the literature for metal–free N–doped carbon catalysts. Zn–air batteries using N–HPC as the electrode material have a higher power density of approximately 40% compared with commercial Pt/C catalysts. Increased efficiency in electrocatalysis is also enhanced by the high *S_BET_* of 3151.2 m^2^/g resulting from the enormous micro–mesoporosity of N–HPC.

The carbon π electrons in B–doped CNs are activated by conjugating with the vacant 2pz orbital of B, activating the B atoms towards electrocatalysis [36]. The B–doped CNOs exhibited higher tolerances for methanol, higher activities, higher stabilities, and lower costs than commercially used Pt/C. They can be a promising candidate for cost–effective ORR [152,153,154]. The authors observed the synergistic effect of two kinds of heteroatom-doping (B– and N–co–doped and P– and N–co–doped CNs), where the electron transfer and reaction energy in ORR and oxygen evolution reaction (OER) were strongly affected by the presence of two types of heteroatoms.

**Table 1:** Some examples of CNs doped by heteroatoms and their short characteristic.

Heteroatom Doping	Substrates	Methods	Carbon Nanostructures	Concentration of Doping Elements	Nature of the Bonding Environment	Significant Properties	Applications	Ref.
Nitrogen	Collagen	Thermal treatment Acid treatment	N–OLC	7.5 at. % N	Pyridinic N Pyrrolic N	Excellent operation stability	ORR catalysis Fuel cells	[114]
	Acetonitrile	Pyrolysis	N–CNO	4.0 at. % N	Pyridinic N Pyrrolic N Graphitic N	Long–term stability	ORR catalysis Nitride sensor	[155]
	Graphene Acetonitrile	CVD	N–Graphene	4.0 at. % N	Pyrrolic N SW defects	High concentrations of defects	Supercapacitor	[55]
	Graphene Melamine	Thermal treatment	N–Graphene	3.7 at. % N	Pyridinic N Pyrrolic N Graphitic N	High current and power density	ORR, OER, HER catalysis	[156]
	Melamine L–cysteine	Polymerization Pyrolysis	N–Graphene nanoribbons	20 at. % N (800 °C) 5.9 at. % N (1000 °C)	Pyridinic N Graphitic N	Bifunctional electrocatalytic activity Excellent cycling stability	ORR and OER catalysis Zn–air batteries	[81]
	GO oxidant	Post-modification	N,O–GON	3.4 at. % N	Pyrrolic N Pyridinic N	High corrosion resistance High selectivity H_2_O_2_	ORR catalysis	[137]
	CNT Ionic liquid	Carbonization Post–modification	CNT/porous carbon (Core–sheath)	4.6 at. % N 1.1 at. % S 5.4 at. % O	Pyridinic N Pyrrolic N Graphitic N	Durability and tolerance towards methanol	ORR catalysis	[157]
	Aminated–ND particles	Thermal treatment	N–CNO	1.0 at. % N	Pyridinic N Pyrrolic N Graphitic N	Homogenous distributions of defects and active N sites	Electrocatalysis Catalytic activity towards H_2_O_2_	[48,134]
	SiO_2,_ DCD, APTES, ZIF–8, Zn(NO_3_)·6H_2_O	Inorganic synthesis Thermal treatment Etching SiO_2_	N–HPC	1.2 at. % N	Monovacancy coupled pyridinic N Graphitic N	Hierarchical porosity *S_BET_* = 3151.1 m^2^/g	ORR catalysis Zn-air batteries	[151]
Boron	ND particles Amorphous boron	Thermal treatment	B–CNO	0.76–3.21 at. % B	Substitutional B (B-C) Planar BC_3_ nanodomain	High crystallinity Excellent long–term charge/discharge stability	Catalysis Electrochemical capacitors	[34,120]
	ND particles Boric acid	Thermal treatment	B–CNO CNO	0.82–3.31 wt. % B	Boron atom cluster B_4_C Substitutional B (B-C)	*σ* = 1.62 1/Ωcm *σ* = 0.80 1/Ωcm	ORR catalysis	[69]
	GO, Boric acid	Ultrasonic treatment Hydrothermal reaction	B–rGO	16.2 at. % B	Substitutional B (B-C) BCO_2_; BC_2_O	The lowest onset potential for B–doped graphene (0.83 V vs. RHE)	ORR catalysis	[158]
Multi-heteroatoms	CNT, IL, silica	Post-modification Thermal treatment	N,S,F–CNT	Total N,S,F 8.8 wt. %	Pyridinic N Pyrrolic N Graphitic N	High durability Superior tolerance towards poisons	ORR catalysis Alkaline fuel cells	[157]
	Solid graphite rod N_2_ atmosphere Amorphous boron	Arc-discharge evaporation	CNT N,B–CNT	- 0.5 at. % N 0.5 at. % B 1 at. % B and 1 at. % N	- Graphitic N N on edges and in topological defects Point substitutional defects C–B and C–N	*σ(T)/σ(T_r_)* = 163 S/cm *σ(T)/σ(T_r_)* = 184 S/cm Charge carriers: 3 × 10^18^ cm^−3^ *σ(T)/σ(T_r_)* = 314 S/cm *σ(T)/σ(T_r_)* = 493 S/cm Charge carriers: 1 × 10^21^ cm^−3^	Conductors Magnetoconductors	[159]
	Melamine Phosphoric acid	Thermal treatment	g–P–C_3_N_4_	2.69–5.0 at. % N 0.40–3.34 at. % P	C defect coupled with N doping	High graphitization Edge defects	ORR catalysis	[160]
	ND Boric acid	Thermal treatment	N–B–CNO	8.0 at. % B 7.4 at. % N	Substitutional B (B–C) BC_3_; BCO_2_; BC_2_O Pyridinic N Pyrrolic N Graphitic N; B–N	High degree of defects	ORR catalysis	[161]

Abbreviations in alphabetical order. APTES: (3–aminopropyl)triethoxysilan; CNC: carbon nanocages; CNO: carbon nano–onion; CNT: carbon nanotube; CVD: chemical vapour deposition; DCD: dicyandiamide, g-C_3_N_4_: graphitic carbon nitride; GO: graphene oxide; GQD: graphene quantum dot; GON: graphene oxide nanoribbons; HER: hydrogen evolution reaction; N-HPC: nitrogen–doped hierarchical porous carbon; IL: ionic liquid; ND: nanodiamond; OER: oxygen evolution reaction; OLC: onion-like carbon; ORR: oxygen reduction reaction; rGO: reduced graphene oxide; ZIF-8: zeolitic imidazolate framework compound. *σ*: electrical conductivity; *σ(T)/σ(T_r_)*: conductivity normalized to room temperature; *S_BET_*: specific surface area determined using BET theory.

Only such atomic configurations of the B and N–pyridinic active sites were preferred, with a small distance between the two atoms. The DFT calculations showed that the chemical coupling of the B and N–pyridinic active sites occurred then, increasing ORR activity. In acidic electrolytes, CNs co–doped with N and S are very promising [36]. The N–C–S–defect–based motifs in carbon materials decreased the energy barriers for ORR in acidic solutions, making the material better than the Pt/C commercial electrocatalyst.

All these factors may consequently lead to an enhancement of the catalytic efficiency due to the increasing of active sites in CNs [81,162,163,164]. The latter results from the doping process using various substrates and methods. We can distinguish thermal treatment at the gaseous atmosphere and reduced pressure [114,136,157], pyrolysis [136], CVD [55], MW–assisted synthesis [165], arc–discharge evaporation [159], etc., (Table 1). Some of these methods are used as one–step processes; others must be supported by multi-step strategies that optimize the reaction conditions. The catalytic activity of CNs is also affected by their morphology, mainly the type of surface determined by their porosity [49]. The size of the pores (micro–, meso– and macropores), their number, and their arrangement affect the efficiency of the catalytic reaction:

i. micropores allow more active sites into the electrolyte;
ii. mesopores can facilitate the mass transport in the catalyst layer;
iii. macropores ensure the catalyst’s long–term stability [49].

**Figure 7:**
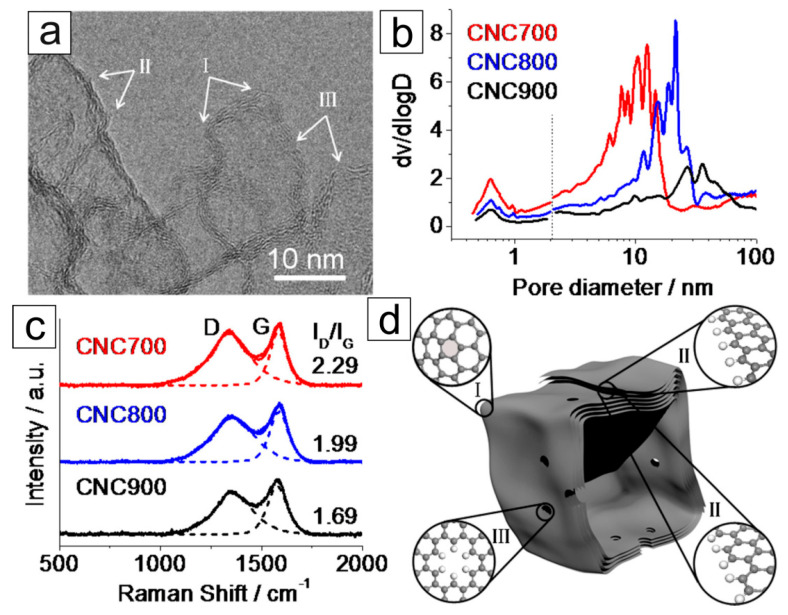
Characterizations and schematic structural characters of the carbon nanocages. (**a**) High–resolution TEM image of CNC700. (**b**) Pore size distributions. (**c**) Raman spectra. *I_D_*/*I_G_* is the area ratio of the D peak to the G peak. (**d**) Schematic structural characters of the carbon nanocages. I, II, and III in panels (**a**,**d**) represent three typical defective locations, i.e., the corner, the broken fringe, and the hole, respectively. Reprinted with the permission from American Chemical Society, Ref. [37].

Zheng Hu and coworkers presented an example of carbon nanocage preparation and the influence of d–CNs on electrocatalytic activity in 2015 [37]. Carbon nanocages were synthesized by the hard-templating method from benzene as the precursor. Next, the pyrolysis results in forming carbon nanocages with a cuboidal hollow structure 10–20 nm in size. This size corresponds to the thickness of the coating from four to seven graphitic layers (Figure 7a). Increasing the temperature from 700 to 900 °C led to an increase in the nanocages’ average size and wall thickness. Temperature also affects the distribution of pores and the number of defects in the structure. Micropores (∼0.6 nm) and mesopores (5–50 nm) coexist in the materials. However, an increase in temperature causes the pore distribution to shift toward the mesopores (Figure 7b) while reducing the SSA from 1713 m^2^/g (700 °C) to 614 m^2^/g (900 °C). As the temperature increases, the crystallinity of the material increases, i.e., the number of defects in the material decreases (Figure 7c). The highest concentration of the defects was detected for the material pyrolyzed at 700 °C. The observed defects in the carbon nanocages are topological disclinations (Figure 7d), in which zigzag edge and pentagon defects are responsible for the electrocatalytic activity of these CNs.

## 4. MW–Assisted Synthesis for the Preparation and Modification of Materials Containing d-CNs

### 4.1. Definition of MW–Assisted Synthesis

**Figure 8:**
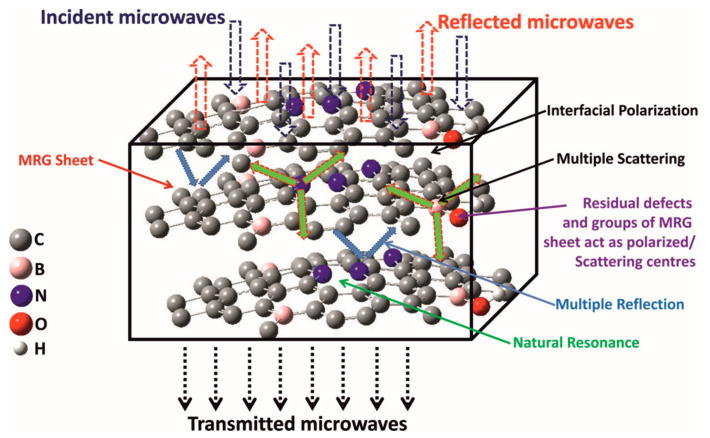
Schematic representation of possible MW absorption mechanism in B– and N–doped porous graphene sample. Reprinted with the permission from American Chemical Society, Ref. [170].

MW irradiation is an influential heating parameter for carbon-based solid materials because they usually absorb electromagnetic radiation well [166,167]. This high–frequency electromagnetic radiation with a wavelength between 0.001 and 1 m (frequencies between 300 and 0.3 GHz) can achieve a temperature of over 1000 °C within a few minutes. Heating of the material is possible due to the interaction of electromagnetic radiation of appropriate energy with charged particles (Figure 8) [168,169]. When polar molecules interact with MW radiation, this causes them to rotate and move, and this, in turn, causes friction. Consequently, the generated energy is dissipated as heat (dipolar polarization). In the case of solid phase materials that are dielectrics, charged particles such as π electrons in carbon materials induce a current flow in the material. In this case, the energy is also dissipated as heat, related to the Maxwell–Wagner effect (interfacial polarization). For the optimal interaction of MW with solid materials, its penetration depth must also be considered [168]. However, it has to be noted that penetration depth becomes a critical factor in micron–scale materials. For the materials in the nanoscale, this problem should not occur.

Significant advantages of using MW heating [167]:

i. contactless heating;
ii. a direct transfer of energy to the reactants;
iii. independence from heat convection;
iv. rapid heating rates, easy control of irradiation parameters;
v. selectivity of heating;
vi. the possibility of conducting the reaction locally and volumetrically.

The MW–based methods may be divided into two groups:

1. Top-down methods, which include the transformation of solid materials into carbon nanomaterials.
2. Bottom-up methods, which include the preparation of carbon nanomaterials from liquid or gaseous carbonaceous precursors.

It is possible to use the energy of the MW irradiation to create new carbon materials and as a method for the purification, functionalization, or annealing of CNs [169]. It is often a method supporting other processes and procedures. Depending on the specific structural properties of the CNs, MW irradiation interacts specifically with the material due to the particular absorption properties of the CNs [171]. The temperature reached in a given process will depend on many parameters. Depending on the type of CNs, their purity (for example, metallic dopants), the defects’ structure, creates functional groups on the surface (for example, containing oxygen), and during the interaction of the carbon material with MW irradiation, local heating may occur. Conversely, when the carbonaceous material is a poor absorber of MW irradiation, it is necessary to add substances to make the MW heating process more efficient [168,172]. In this case, substances such as polymers, conducting materials or ionic materials, metallic nanoparticles, etc., should be applied [173,174,175].

### 4.2. MW-Assisted Synthesis for the Preparation and Modification of d–CNs: Implications on Properties and Applications

MW-assisted synthesis is a powerful heating method for preparing or modifying carbon-based solid materials [176]. Currently, this method is most often used to design and modify graphene (G), carbon nanotubes (CNTs), carbon quantum dots (CQDs), and graphitic nitride carbon (g–C_3_N_4_). By MW–assisted heating with the different carbon sources (graphite [177,178], metallocenes [171,178], carbon nanoparticles [179], polymers [180,181], etc.) in the presence of a catalyst and in the gas phase, different CNs were synthesized (Table 2). 

Non–modified CNs have somewhat limited charge accumulation properties [182,183,184]. To optimize the electrochemical properties of carbon materials, they are combined with redox capacitive materials, which are involved in faradaic reactions. Combining the mechanical and electrochemical stability of CNs and their high SSA resulting from micro-mesoporosity produces hybrid materials with excellent electrochemical properties. Examples of the hybrid materials synthesized using MW irradiation are listed in Table 2, and their specific parameters determine subsequent applications. The optimized electrochemical properties of the mixed materials are mainly due to the possibility of creating mesoporous inorganic structures with different structural characteristics (amorphous and crystalline materials) combined with highly conductive CNs. Depending on the experimental conditions of the MW synthesis, it is possible to create materials with a different degree of structure organization with a higher degree of crystallinity, leading to obtaining composites with better electrochemical properties. For example, an rGO/Fe_3_O_4_ composite was obtained in which the Fe_3_O_4_ inorganic phase was in the form of nanoparticles or octahedral nanocrystals (Table 2) [185]. In this case, the organization of Fe_3_O_4_ into octahedral crystals on the rGO surface increased the *C* value from 1050 to 1625 mA h/g at a current density (*j*) of 100 mA/g.

**Table 2:** Some examples of using MW–assisted synthesis for the preparation and modification of pristine d–CNs and hybrid materials containing d–CNs.

Defective CNs	Materials Containing d–CNs	Methods	Experimental Conditions	Applications	Significant Properties	Refs.
Pristine d-CNs	d–G (hydrogel)	MW–assisted hydrothermal synthesis	*P* = 800 W; *t* = 5 min	Supercapacitors	*C_s_* = 340 F/g (*j* = 0.5 A/g)	[186]
	N,S–GO	MW–assisted synthesis	*P* = 800 W; *t* = 5 min	Supercapacitors Universal: aqueous, non–aqueous, ionic electrolytes	*C_s_* = 460 F/g (*j* = 1 A/g) *C_s_* = 810 F/g (*j* = 3 A/g)	[118]
	S–rGO	MW–assisted synthesis	*T* = 140 °C; *t* = 30 min	Supercapacitors	*C_s_* = 238 F/g	[187]
	rGO N,B–rGO	Chemical synthesis MW–assisted synthesis	*P* = 700 W; *t* = 40 s	EMI shielding devices	*σ* = 21.4 S/m *σ* = 124.4 S/m	[170]
	rGO (porous)	MW–assisted synthesis	*P* = 700 W; *T* = 180 °C; *t* = 6 min	Supercapacitors	*C_s_* = 568 F/g (*j* = 1 A/g)	[188]
	rGO	IL–assisted MW synthesis	*P* = 700 W; *t* = 15 s	Supercapacitors	*C_s_* = 135 F/g; *E_m_* = 58 Wh/kg; *P_m_* = 246 kW/kg	[189]
	d–CNT	MW hydrogen plasma processing	*P* = 200 W *t* = 30 and 60 min	Vacuum electron sources	*j_emission_* = 10.36 mA/cm^2^	[106]
	GO/g–C_3_N_4_	Ultrasonic–MW–assisted synthesis	*P* = 700 W; *t* = 5 min	Photocatalytic H_2_ evolution	Photocatalytic H_2_-production rate 224.6 μm/h; GO electron collector and transporter	[190]
	GO/g–C_3_N_4_	MW–assisted synthesis Chemical treatment	*P* = 700 W; *t* = 5 min	Supercapacitors	*S_BET_* = 353 m^2^/g; *C_s_* = 113 F/g *S_BET_* = 686 m^2^/g; *C_s_* = 169 F/g	[191]
	N–PGF	MW–assisted synthesis	*P* = 800 W; *t* = 4 s	Supercapacitors	*C_s_* = 272.6 F/g; *E* = 2.3 mW h/cm; *P* = 0.42 W/cm	[192]
Hybrid materials containing d-CNs	CNT/Fe_2_O_3_	CVD; MW hydrothermal synthesis	*T* = 160 °C; *t* = 6 h	Lithium–Ion Battery Electrodes	*C* = 900 mAh/g	[193]
	CNT/NiMn_2_O_4_	MW–assisted hydrothermal synthesis	*P* = 800 W; *T* = 160 °C; *t* = 1 h	Supercapacitors	*C_s_* = 916 F/g (*j* = 1 A/g) *E_m_* = 36.5 Wh/kg; *P_m_* = 800 W/kg	[194]
	MWCNT/CoMoO_4_	MW–assisted solid-state synthesis	*P* = 480–720 W; *t* = 8 min	Supercapacitors	*C_s_* = 170 F/g (*j* = 0.1 A/g)	[195]
	NiS@CNT/NiO	MW–assisted solid-state synthesis	*P* = 1000 W; *t* = 60 s	Supercapacitors	*C_s_* = 810 F/g (*j* = 1 A/g)	[196]
	rGO/NiS	MW–assisted hydrothermal synthesis	*P* = 700 W; *t* = 4 min	Supercapacitors Solid–state Supercapacitors	*C_s_* = 1746 F/g (*j* = 1 A/g) *C_s_* = 14.20 F/g; *E_m_* = 7.1 Wh/kg; *P_m_* = 1836 W/kg	[197]
	rGO/Fe_3_O_4_ NPs rGO/Fe_3_O_4_ ONCs	Chemical exfoliation MW–assisted synthesis	*P* = 700 W; *t* = 1.25–1.75 min	Lithium–Ion Battery Electrodes	*C* = 1050 mA h/g (*j* = 100 mA/g) *C* = 1625 mA h/g (*j* = 100 mA/g)	[185]
	rGO/CNT/Ni_NP_	Thermal exfoliation MW–assisted synthesis	*P* = 700 W; *t* = 5 min	Lithium–Ion Battery Electrodes	*C* = 648.2 mA h/g (*j* = 100 mA/g) *C* = 282.4 mA h/g (*j* = 100 mA/g)	[198]
	rGO/NiO/Co_3_O_4_	MW–assisted synthesis	*P* = 700 W; *t* = 45 s	Supercapacitors	*C_s_* = 910 F/g (*v* = 20 mV/s) *S_BET_* = 99.5 m^2^/g	[199]
	rGO/CoAl–LDH	MW–assisted reflux synthesis	*P* = 1000 W; *T* = 100 °C; *t* = 2 h	Supercapacitors	*C_s_* = 772 F/g (*j* = 1 A/g) *E_m_* = 22.7 Wh/kg; *P_m_* = 230 kW/kg	[200]
	rGO/NiAl–LDH	MW–assisted reflux synthesis	*P* = 1000 W; *T* = 100 °C; *t* = 2 h	Supercapacitors	*C_s_* = 1630 F/g (*j* = 1 A/g) *S_BET_* = 121.2 m^2^/g	[201]
	rGO/NiMoO_4_	MW–solvothermal synthesis Thermal annealing	*P* = 200 W; *T* = 115 °C; *t* = 25 min	Supercapacitors	*C_s_* = 1274 F/g (*j* = 1 A/g) *S_BET_* = 50.8 m^2^/g *C_s_* = 800 F/g (*j* = 1 A/g) *S_BET_* = 30.9 m^2^/g	[202]
	N–G/NiS	MW–assisted synthesis	*P* = 336 W; *t* = 18 min	Supercapacitors	*C_s_* = 1468 F/g (*j* = 1 A/g) *E_m_* = 66.6 Wh/kg; *P_m_* = 405.8 W/kg	[203]
	rGO/MnCo_2_O_4_	Exfoliation MW–assisted synthesis	*P* = 900 W; *t* = 45–70 s	Supercapacitors	*C_s_* = 562 F/g (*v* = 20 mV/s)	[204]
	G/α–MoO_3_	MW–assisted synthesis	*P* = 700 W; *t* = 7 min	Supercapacitors	*C_s_* = 483 F/g (*j* = 1 A/g)	[205]
	rGO/CoSe_2_	MW–assisted synthesis Thermal annealing	*P* = 700 W; *t* = 7 min	Supercapacitors LED	*C_s_* = 761 F/g (*j* = 1 A/g) *E_m_* = 43.1 Wh/kg	[206]
	G/Co_9_S_8_	MW–assisted hydrothermal synthesis	*P* = 700 W; *T* = 160 °C; *t* = 30 min; *p* = 8 × 10^6^ Pa	Supercapacitors	*C_s_* = 1150 F/g (*v* = 5 mV/s)	[67]
	rGO/MnN	MW–assisted synthesis	*P* = 900 W; *t* = 1 min	Sodium ion batteries Supercapacitors	*C* = 16 mAh/g *C_s_* = 639.2 F/g (*v* = 10 mV/s)	[207]
	rGO/MnO_2_	Conventional synthesis MW–assisted synthesis	*P* = 700 W; *t* = 2 min; 21 cycles	Supercapacitors	*C_s_* = 140 F/g (*j* = 1 A/g)	[208]
	rGO/Pd	MW–assisted synthesis	*P* = 810 W; *t* = 90–125 s	Electrocatalysis (Ethanol Oxidation)	Catalytic activity 10.2 mA/cm^2^ (Pd)	[209]
	S–rGO/NiFeS_2_	MW–assisted synthesis	*P* = 800 W; *t* = 3 min	Supercapacitors	*C_s_* = 1073 F/g (*j* = 1 A/g) *E_m_* = 45.7 Wh/kg; *P_m_* = 222 W/kg	[210]
	3D Pd–E–PG	MW–assisted synthesis	*P* = 700 ÷ 900 W; *t* = 30 ÷ 60 s	H_2_ storage CO oxidation	H_2_ 5.4 wt. % 100% CO conversion < 300 °C	[211]
	NiF–G/SimonK	MW–hydrothermal synthesis	*P* = 700 W; *t* = 1 h; *p* = 100 bar	Supercapacitors	*C_s_* = 836 F/g (*j* = 1 A/g)	[212]
	G/NiCoS	MW–assisted synthesis	*P* = 600 W; *t* = 20 min	Supercapacitors	*C_s_* = 1186 F/g (*j* = 1 A/g) *E_m_* = 46.4 Wh/kg	[213]
	G/CNT/Pd	IL–assisted MW synthesis	*P* = 700 W; *t* = 10 min	Energy storage systems	*C_s_* = 1615 F/g (*v* = 10 mV/s)	[214]
	CMK-3/CNT	Hard–templating method MW–assisted synthesis	*P* = 700 W; *t* = 30 s	Supercapacitors	*C_s_* = 315 F/g (*j* = 1 A/g)	[68]

Abbreviations in alphabetical order. CMK–3: ordered mesoporous carbon; CNT: carbon nanotubes; CQD: carbon quantum dot; d–CNT: defective carbon nanotube; DSSC: dye–sensitized solar cells; EMI: electromagnetic interference; G: graphene; GO: graphene oxide; rGO: reduced graphene oxide; g–C_3_N_4_: graphitic carbon nitride; LDH: layered double hydroxide; LED: light emitting diode; MnN: manganese nitride; MWCNT: multi–walled carbon nanotube; NP: nanoparticle; N-PGF: nitrogen–doped porous graphene framework: NCS: nickel cobalt sulfides; NiF: nickel foam; ONC: octahedral nanocrystal; ox-CNT: oxidized carbon nanotubes; *o*–PDA–co–PANI: copolymer of *ortho*–phenylenediamine and aniline; 3D Pd–E–PG: Pd–embedded three dimensional porous graphene; SimonK: Simonkolleite (Zn_5_(OH)_8_Cl_2_·H_2_O). Physical quantities: *σ*: electrical conductivity [S/m]; *E_m_*: specific energy density [Wh/kg]; *j*: current density [A/g]; *p*: pressure; *P_m_*: specific power density [W/kg]; *P*: power [W]; *T*: temperature [°C]; *t*: time; *S_BET_*: specific surface area determined by N_2_ adsorption method using BET theory.

Although for most hybrid systems, the SSA values determined usually have low values below 100 m^2^/g, the synthesized materials show superior capacitive performance (Table 2). Firstly, the addition of CNs to the inorganic phase decreases the degree of aggregation of the inorganic material. The total capacity of the electrode is related to two charging mechanisms: electric double–layer charging (EDLC) and pseudocapacitance [130,215]. The first process is related to the presence of carbonaceous material in the structure, and, to put it simply, the capacitance of the EDLC depends on the SSA [216]. The second mechanism is based on the redox reaction of the inorganic material, mainly transitional metal oxides and multiplex systems with stoichiometric and nonstoichiometric compositions [215]. Since the percentage of inorganic components in the composite is usually several times higher than CNs, this mechanism is dominant in hybrid electrodes [217]. As a result, in composite electrodes, in which there is good contact between the organic and inorganic phases, and their homogeneous dispersion is ensured, the communication between electrolyte ions and active electrochemical materials is facilitated, and the ion diffusion path is shortened. It is essential to point out that the presence of CNs in the composite decreases the material’s resistance and increases its cycling stability as well as energy density (*E*) and power density (*P*) compared with pristine inorganic materials [217]. Table 2 summarizes examples of d–CNs and hybrid systems obtained using MW irradiation, indicating the materials’ reaction parameters and critical properties.

**Figure 9:**
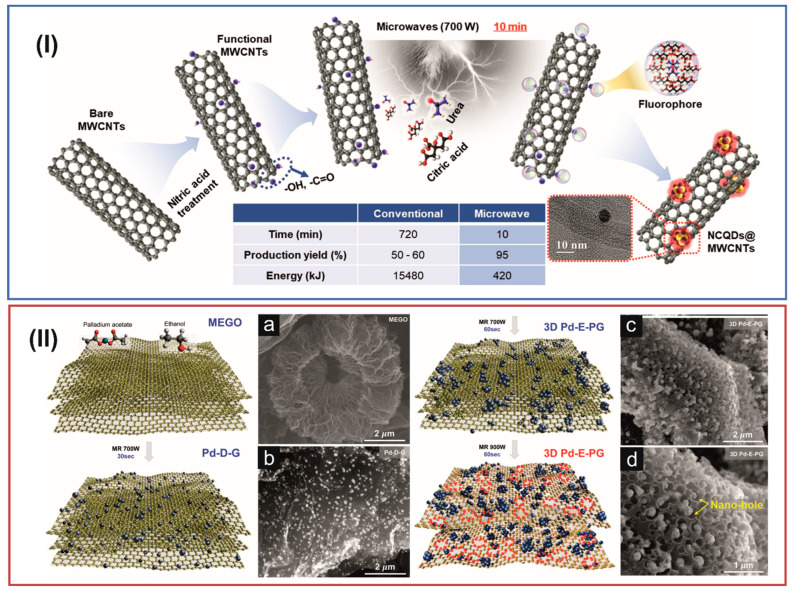
Panel (**I**): Schematic illustration of the synthesis of MWCNT/N–CQD, where mild oxidation generates active sites for the growth of N-CQDs. The inset table compares the conventional synthesis with the MW–assisted process. Reprinted with the permission from Elsevier, Ref. [218]. Panel (**II**): Synthesis route of nanohole-structured and Pd–embedded 3D porous graphene (3D Pd–E–PG) and corresponding SEM images. Schematic illustration of the MW fabrication process of the 3D Pd–E–PG. (**a**) SEM image of MW–exfoliated graphene oxide. (**b**) SEM image of the uniform decoration of Pd nanoparticles on graphene layers after low–power MW irradiation. (**c**) SEM image of the aggregation of Pd nanoparticles after successive high–power MW irradiation. (**d**) SEM image of nanohole generation and the perforated graphene structures after multistep MW irradiation. Reprinted with the permission from American Chemical Society, Ref. [211].

Figure 9 presents two examples of MW irradiation’s use for synthesizing different compositions of carbon-based materials. Panel (I) shows the preparation of N–doped carbon quantum dot (N–CQD)/MWCNT material using the conventional method and MW–assisted synthesis [218]. The first stage of the process was based on the most popular method of MWCNT oxidation using concentrated nitric acid. As a result of this reaction, oxygen–containing functional groups were introduced on the MWCNT surface. In the further stage of the reaction, these sites (structural defects) are active for further functionalization. Further, in the presence of urea and citric acids and under heating, N–CQDs are formed on the surface of MWCNTs (Figure 9I). The authors of this work also carried out, for comparison, the stage of N–CQD production under conventional reaction conditions. The data summarized in Table 2 indicate that using MW irradiation to synthesize N–CQDs shortens the reaction time and energy consumption and increases the efficiency of the reaction. Figure 9II shows the possibility of the multifunctional use of MW irradiation, depending on the power and duration of its use (see also Table 2). Kumar and colleagues used the MW irradiation process to obtain nanohole–structured and Pd–embedded 3D porous graphene (3D Pd–E–PG) [211]. The process was multi–stage. The first stage was graphene’s exfoliation and the addition of ethanol and Pd acetate to the reaction mixture. Then, under the influence of MW irradiation with a power of 700 W, within 30 s, Pd nanoparticles were created. Next, MW irradiation with a power of 700 W for 60 s was applied again, which caused the formation of a 3D structure consisting mainly of the agglomeration of Pd nanoparticles. In the last stage of the process, a higher power MW irradiation (900 W, 60 s) was used, which caused the perforation of the graphene layer (creation of defects). Holes of a size analogous to Pd nanoparticles enabled the distribution of nanoparticles in the entire volume of the carbon material, creating a compact and interconnected structure of 3D Pd–E–PG. The course of each stage was confirmed using scanning electron microscopic (SEM) imaging (Figure 9II(a–d)). Figure 9II(a) shows the graphene layers, which, resulting from MW irradiation, form a brush–like and wrinkled structure. In the next stage, the Pd nanoparticles are evenly distributed on the graphene layers (Figure 9II(b)). As a result of the MW irradiation of a specific time and power, the graphene layers are covered with a large amount of Pd nanoparticles. Further irradiation leads to the formation of nanoholes (10–100 nm) (Figure 9II(c,d)), in which metallic nanoparticles are placed, creating a 3D structure of Pd-E-PG, with the simultaneous formation of increasingly larger metallic agglomerates.

Generally, the synthesis or modification of carbon–based materials using MW irradiation follows the presented paths. In some cases, running the reaction in multiple steps is necessary, mainly to increase the yield of the reaction. Despite this, the procedures are much more straightforward, thanks to the short reaction times under MW–assisted heating. In addition, in the case of these methods, the purification process is much simpler.

Graphene is a 2D atomic crystal with extreme mechanical strength and high electronic and thermal conductivities [219]. Due to these outstanding properties, it has been one of the most frequently used CNs in scientific research in recent years [220]. In the case of this carbon structure, MW irradiation is not used to prepare it in bottom–up processes. However, due to its simplicity, MW irradiation modifies a graphite precursor. Exfoliation is therefore possible, yielding graphene oxide (GO) [221]; the reduction of GO [186], resulting in reduced GO (rGO) [188]; the doping of graphene layers with heteroatoms [118,187,189], or a combination of two kinds of CNs in one material [190,192].

**Figure 10:**
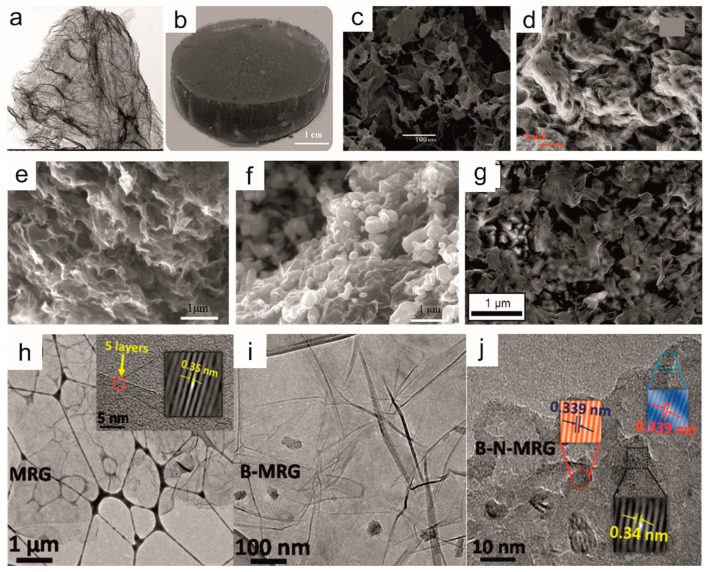
SEM image of (**a**) functionalized graphene [186]. (**b**) Photograph of gelatin–GO aerogel [221] and (**c**) SEM image of gelatin–GO aerogels [221]. SEM images of (**d**) 1:1.5 nickel–cobalt sulfides [118], (**e**) porous rGO (GO:ZnO = 1:4) [188], (**f**) rGO/ZnO (GO:ZnO = 1:4 (without HCl)) composites [188], (**g**) S–rGO–0.25 [187]. Reprinted with the permission from Elsevier, Refs. [186,187,188,221]. (**h**–**j**) TEM images of (**h**) reduced graphene oxide (MRG), (**i**) B–doped MRG (B–MRG), and (**j**) high–resolution TEM image of B– and N–doped MRG (B–N–MRG). Reprinted with the permission from American Chemical Society, Ref. [170].

MW irradiation is often used to functionalize graphene layers [222]. The controlled creation of defects in graphene-based materials is a promising strategy to tailor the electrical, electrochemical, and electrocatalytic properties. For example, a hydrogel was obtained from oxidized graphene during MW–assisted synthesis in hydrothermal conditions (Figure 10a) [186]. This one–step process resulted in a 3D carbon structure resembling crinkled paper. Such a 3D graphene structure can accelerate the transport and diffusion of ions, and functional groups reduce the aggregation of graphene layers. The obtained material exhibits a high specific capacitance (*C_S_*) of 340 F/g at *j* of 0.5 A/g and excellent stability with approximately 97.3% retention of the initial *C_S_* after 20,000 cycles (at 10 A/g) (Table 2). These parameters indicate that d-G (hydrogel) may be a promising electrode material for high–performance SCs.

Analogous conditions were also used to obtain GO-based gelatin aerogels (Figure 10b,e) [221]. The binary aerogel was studied under acidic and alkaline conditions. Increasing the gelatin content in aerogel raises the modulus of elasticity to 6–fold and the swelling ratio up to 1.4–fold. The properties of the aerogel obtained using MW irradiation were compared with this synthesized using conventional heating. Similar physicochemical properties of both aerogels were identified; however, the swelling ratio value was 1.5 times higher for the aerogel synthesized using MW irradiation.

The following example shows the possibility of using the MW–assisted method to produce porous reduced graphene oxide (rGO) (Figure 10e,f) [188]. Conducting the synthesis under MW irradiation conditions causes a shorter period and lower temperature, employing hydrochloric acid as an etching agent. In addition to lowering energy consumption, preparing the material under these conditions leads to obtaining material that avoids the restacking of subsequent rGO layers and numerous pores and promotes material transport efficiency. It is a critical phenomenon because reduced GO is characterized by a strong stacking effect of individual graphene layers, significantly limiting the potential of using this material in devices that accumulate electric charge. The combined macro/mesopore effect in porous rGO provides accessible ion transport pathways for the base electrolyte compared with conventionally prepared rGO. The electrochemical studies show that the synthesized rGO’s calculated *C_S_* was 568.5 F/g at 1 A/g with a remarkable capacity retention after longer charge/discharge cycles (Table 2).

An exciting example of using MW irradiation to modify GO is the method presented by Kwang S. Suh et al. [189]. The proposed facile and scalable method leads to the production of rGO by ionic liquid (IL)–assisted MW chemistry. ILs were used as sources of dipoles and the doping element rGO. The resulting material is characterized by the open porous architecture of rGO filled with IL moieties, resulting in easy ion transportation and consequently exhibiting a high *C_S_* of 135 F/g. Additionally, a device operated at a voltage of 3.5 V revealed a high *E*∼58 Wh/kg and *P* amounting to 246 kW/kg.

MW radiation can easily be adapted to modify the graphene structure so that the carbon atoms in the graphene layer are replaced with other heteroatoms, such as N, S, P, or B [118,119,187] (please see Table 2). Deepak K. Pattanayak and coworkers presented an interesting example in 2020 (Figure 10d) [118]. Using a one–pot MW–assisted synthesis resulted in the doping of graphene with a high amount of N and S atoms (N,S–GO). The CN’s heteroatom content, at 14.9% of N and 4.3% of S, leads to very high *C_S_* values of 310 F/g in two electrodes symmetric configuration (1 M H_2_SO_4_ electrolyte). N,S–GO was also used in non–aqueous organic and IL electrolytes, where N,S–GO shows *C_S_* of 226 F/g and 150 F/g with an energy density of 32 Wh/kg, respectively. The explanation of the heteroatom–doped graphene layer and the effect on the change of physical properties are described in detail in Section 3. Here, we will limit ourselves to discussing only some literature examples of preparing this doped structure.

Doping heteroatoms are an effective way to modify the physical properties of rGO. The modification of rGO using MW–assisted synthesis was applied to prepare S–doped rGO with different concentrations of S (Figure 10g) [187]. The synthesis was performed in mild experimental conditions at 140 °C for 30 min, which led to the wrinkling and folding of graphene sheets. The material with the highest content of S shows excellent electrochemical performance. A 5–fold increase in the number of sulfur atoms in the graphene sheets leads to a rise in the value from 61.7 to 237.6 F/g. The synthesized material has a promising potential for SC applications with simultaneous high electrochemical stability and capacitance retention of 106% after 10,000 cycles.

MW–assisted synthesis was also used to prepare B– and N–doped; and B,N–doped rGO (Figure 10h–j) [170]. The synthesized material possesses electromagnetic interference (EMI) shielding properties and high electrical conductivity. B,N–doped rGO shows high electrical conductivity compared with other materials: rGO, N–doped rGO, and B–doped rGO, which results in better EMI shielding ability. A high EMI shielding of −42 dB (∼99.99% attenuation) for B,N–doped rGO was measured compared with undoped rGO (−28 dB). The electrical conductivity increases from 21.4 (rGO) to 124.4 S/m (B,N–doped rGO) due to the nanojunction inside the material. The electrical conductivity depends on the temperature. In a low-temperature range (*T* < 50 K), the mechanism of the electrical conductivity is based on 2D–variable range hopping and the Efros–Shklovskii–VRH conduction model. 

ND powders with different average particle sizes (10 nm to 1 μm) are sensitive to MWs between 2.49 and 9.43 GHz [223]. Increased permittivity with decreasing particle size, polarization, and MW loss has already been observed. The oxygenation and hydrogenation of the NDs (sp*^2^*–hybridization was raised in the sample) led to dielectric polarization, and the loss increased [172]. Various methods have been applied to increase the heating rate further and induce demanding reaction conditions. For example, specific substances, coating (MW–transparent substrates) or triggers have been added to tune the reaction conditions [167,169,172]. Using NDs in the environment of conducting polymers (ND–polyaniline) in MW–assisted synthesis significantly enhanced the MW absorption due to additional and intense polarization originating from the HN–CO groups acting as asymmetric centers [173]. These studies showed that ND particles might be applied as MW absorbers.

In some cases, such synthesis may require the addition of easily polarizing substances to tune the reaction conditions and to increase temperature. MW–assisted synthesis was also applied for the preparation of onion-like structures in the heavy crude oil and carbon catalyst (activated carbon and NiO–MoO_3_/γ–Al_2_O_3_; 1:1 *w*/*w*) and tomatoes/carrots as a source of carbon and 30% NaOH [224,225]. The obtained CNs were non–homogenous with structural imperfections and an empty core; their structure did not resemble ND–derived CNOs.

The MW–assisted method was also successfully used to prepare and modify other CNs. An IL–assisted splitting method using MW irradiation as an external energy source produced graphene nanoribbons from MWCNT or SWCNT [226]. The process was based on two strategies: oxidation with strong acids and reduction with hydrazine. The MW–assisted method leads to splitting and expanding tubular graphite nanofibers, which consequently results in the preparation of graphene nanoribbons in the hundred nanometers. The MW–assisted synthesis was employed to prepare CNTs from acetylene and hydrogen as precursor gases [227]. The obtained CNTs were used as a sorbent to remove crystal violet. The sorption capacity was 81% upon contact with the analyte for 25 min at pH 7.0 using a sorbent concentration of 10 mg/L.

Using MW irradiation, it was possible to modify the tube walls in such a way as to remove the capped parts of the CNTs (Figure 2 and Table 2) [106]. The morphology of the CNTs was controlled by adjusting the MW powers. MW irradiation led to the opening of the nanotubes and the removal of fragments of the outer layers of CNTs. Moreover, it led to the formation of sp^3^–hybridized defects in the nanostructure. Such a modified structure of CNTs may facilitate the tunneling of electrons through the barriers and increase emission at new active sites.

MW–assisted synthesis was applied to grow N–CQDs on the surface of MWCNTs [218]. In conventional methods, N–CQDs are incorporated into the MWCNT surface by multistep processes, including the synthesis of the N–CQDs, their complex purification, surface activation, and crosslinking with the MWCNT surface. The method is simplified using MW irradiation by direct MW–assisted growth of N–CQDs on the MWCNT surface. Additionally, this surface modification method of MWCNTs effectively modulated their surface reactivity and internal band structure, which has a significant impact when studying electrocatalytic and photovoltaic processes. Based on the hybrid N–CQDs/MWCNT material, the dye–sensitized solar cells showed 50% higher photovoltaic efficiency than MWCNTs.

MW irradiation was applied to synthesizing composite containing GO and graphitic carbon nitride (g–C_3_N_4_), and the material’s photocatalytic activity was tested [190]. A 12-fold increase in the photocatalytic efficiency of GO/g–C_3_N_4_ under visible light irradiation (224.6 μmol/h) in the production of H_2_ compared with g-C_3_N_4_ was observed (Table 2). This effect is due to the ability of GO to accept and transport electrons from the excited g–C_3_N_4_, which significantly influenced the phenomenon of charge separation. The studies show the promising potential of this nanocomposite for electron collectors in photocatalytic H_2_ production.

Gengchao Wang et al. presented an example of MW irradiation, where N–doped porous graphene frameworks were synthesized [192]. It is another example of creating a composite of graphene and CQDs (Table 2). An N–doped porous graphene framework is synthesized quickly during several processes running simultaneously. A readily dispersible graphene was an effective receptor for MW absorption and initiated GO reduction. Next, as an MW absorbing receptor, the reduced part of GO reduces the chain and allows N–doped porous graphene framework formation. The synthesized material has an outstanding electrochemical performance and a volumetric absorption capacity that can be used in catalysis, energy storage, and environmental protection (Table 2). The electrodes made of GO/g–C_3_N_4_ delivered a volumetric *E* of 12.3 mWh/cm^3^ at *P* of 0.42 W/cm^3^. The N–doped porous graphene frameworks exhibited an extremely high volumetric absorption capacity of 100–243 g/cm^3^ for different oils and organic solvents.

These studies showed that CNs might be applied as MW absorbers in MW–assisted synthesis. In some cases, such synthesis may require the addition of easily polarizing substances to tune the reaction conditions and to increase the temperature to achieve the activation energy of a chemical reaction. Optimizing these parameters allows for the synthesis or modification of the CNs quickly.

### 4.3. MW-Assisted Synthesis for the Preparation of Hybrid Materials Containing d–CNs: Implications on Properties and Applications

MW irradiation is also a precious energy source for the preparation of multicomponent systems, often when conventional synthesis is highly complicated due to its multi–stage nature and the product purification being time–consuming. In addition, it is possible to conduct the synthesis in different experimental conditions, such as in the presence of a solvent or the solid phase, in ILs or using materials of various origins, including inorganic and organic. Combining materials into larger supramolecular systems or composites is simple by using MW irradiation due to a short time and low energy consumption.

An ultrafast MW process allows the synthesis of a carbon composite with the ordered mesoporous carbon as the core and CNT as the shell [68]. A 10–30 s MW irradiation catalyzes in situ CNTs growth within the nanochannel of ordered mesoporous carbon (Table 2). The whisker morphology of the obtained composite looks like a rambutan. Such interconnection between CNTs and mesoporous carbon particles effectively bridges 3D conducting networks, promoting the ion adsorption and diffusion of the supporting electrolyte. A composite consisting of a copolymer and CNTs was also prepared [228]. The oxidized CNTs were incorporated into the polymer matrix consisting of poly(*o*–phenylenediamine–co–aniline) using MW irradiation (for 45 min at intervals of 5 min) to accelerate the polymerization. The oxidation of CNTs was also supported by MW irradiation for 30 min at 160 °C. Combining these synthetic procedures led to the preparation of a composite with a needle–like structure of the copolymer. The system of the copolymer and nanocomposite enables the effective accumulation of electric charge. They exhibited a high *C_S_* of 147.14 F/g at 0.50 A/g with a capacitance retention of 82%.

MW irradiation is more often used to combine materials of different chemical natures, mainly for the synthesis of composites containing CNs and metallic nanoparticles [197,204,205,211,213,229,230], nanopellets [212], or core–shell structures [194,195,196]. Metallic nanoparticles can, of course, consist only of pristine metallic nanoparticles [211,214]; they can form metallic connections of various elements [194,195], metal hydroxide [231], metal oxides [185,232], nitride [207], or sulfides [197,210], in various combination, etc.

Since then, the worldwide studies of electrocatalytic processes have been dominated by precious–metal–based–materials: Pt–, Pd–, Mo–, Fe–, and Co–based catalysts for ORR and HER [233,234,235]. Au- and Ag–based catalysts for electrocatalytic CO_2_ reduction reaction (CO_2_RR) [236,237], and Ru– and Ir–based catalysts for OER [238,239]. Generally, metal–based catalysts are characterized by low selectivity, poor durability, and susceptibility to gas poisoning. Due to the cost and the scarcity of some metals, their practical and large–scale use still needs to be improved. Pt nanoparticles have long been regarded as the best catalyst for ORR. However, due to the notable disadvantages listed above, the commercialization of these technologies needs to be improved. Therefore, it is necessary to search for alternative earth–abundant materials. CNs demonstrate excellent electrocatalytic activity with high stability [37,81,240]. A new generation of electrocatalysts is developed, in which defective surface of materials promotes multiple–proton–coupled electron transfer and mass transportation [41]. In this context, combining materials of different chemical nature can optimize the properties of the designed materials.

**Figure 11:**
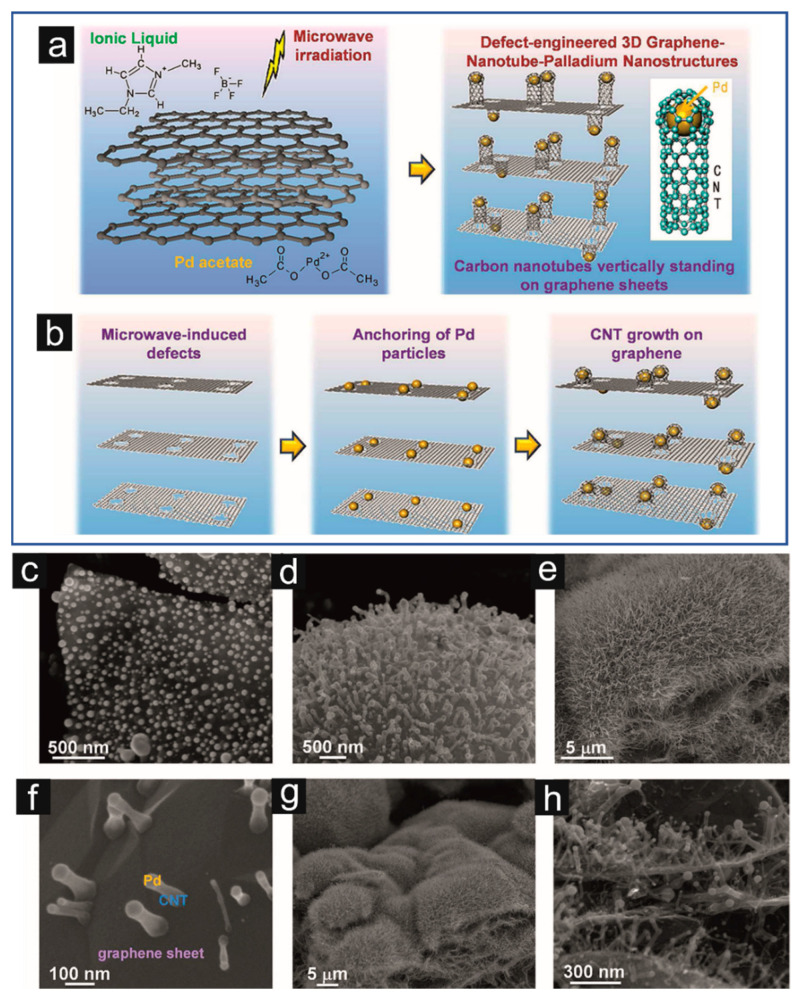
Schematic representation of one–pot MW synthesis of 3D carbon hybrid nanostructures showing vertically grown CNTs on graphene sheets: (**a**) scheme and (**b**) mechanism. (**c**–**h**) SEM images of 3D CNs showing CNTs vertically attached to graphene sheets: (**c**) Pd nanoparticles initially anchored on graphene, (**d**) vertically standing CNTs grown on graphene by Pd nanoparticles, (**e**) CNT forest extensively grown on graphene surfaces after lengthy MW irradiation, (**f**) direct bonding between CNTs and graphene, (**g**) mass production of 3D G–CNT–Pd nanostructures, and (**h**) terrace structures of hybrid material. Reprinted with the permission from American Chemical Society, Ref. [214].

The idea of using MW irradiation in composite preparation containing metallic nanoparticles and CNTs is schematically presented in Figure 11 [214]. In the first stage, CNs with metallic precursors and substances that are good MW absorbers are placed simultaneously in an MW reactor (Figure 11a). Graphene platelets in ILs and Pd acetate were mixed in this case. The impregnation and partial exfoliation of graphene happened due to MW irradiation and weak Van der Waal’s and π–π interaction. The IL reduced the diameter of dissolved Pd cations. As a result, they created Pd nanoparticles, which were distributed on graphene sheets. At the same time, structural defects are formed in the graphene layer due to MW irradiation or the catalytic activity of the IL. These defects act as nucleation sites for core–shell structures of the Pd nanoparticles with imidazolium shells. During further MW irradiation, the outer organic layer decomposes, resulting in it being formed carbonaceous gasses. These are carbon sources for CNT growth. Carbon diffuses on the Pd nanoparticle’s surface and comprises multi–walled core–shell nanoparticles.

It has to be noted that direct bonding between CNTs and graphene was detected due to the defect–based growth mechanism (Figure 11b). Under these conditions, a dense brush of CNTs with Pd nanoparticles placed perpendicularly to the graphene surface was performed (Figure 11d). By manipulating the amount of graphene used for Pd and MW irradiation power and time, the composition of the resulting hybrid material was quantitatively controlled (Figure 11e). In many cases, it is simplified, mainly when we want to get two–component composites with a random 3D organization. This type of functionalization was presented in works in which the carbonaceous component was graphene [211] and N–doped GO [230]. This process is illustrated in detail in Figure 3 and discussed in Section 4.2 [211]. The results show the 3D Pd–E–PG nanostructure has a ∼5.4 wt. % H_2_ storage capacity under 7.5 MPa and CO oxidation catalytic activity at 190 °C. The synthesized N–GO/Pd material was also used as a catalyst for ethanol electrooxidation with a *j* of 10 mA/cm^2^ [230]. Analogous N–rGO/Pd material was used in direct-ethanol fuel cells [209]. The electrocatalytic activity of N–rGO/Pd was accessed by cyclic voltammetry (CV) in the presence of ethanol. The N–rGO/Pd catalyst exhibits better electrocatalytic performance than rGO/Pd, with an electroactive surface area of 6.3 m^2^/g and ∼3.7 m^2^/g, respectively.

In addition to composites containing pure metallic nanoparticles and bimetallic nanoparticles, other compounds containing a metallic component are often synthesized (Table 2). Most commonly, this may include metal oxides [185,204,205,229,231,232], nitride [207], or sulfides [197,210], in various combinations, etc. MW–assisted synthesis is an effective method for the facile and fast preparation of hybrid materials. It is usually the last stage of the synthesis, often preceded by obtaining CNs or their initial modification. A good example is an article on preparing a composite containing MWCNTs and Fe_3_O_4_ (Table 2) [229]. The pristine MWCNTs and N–doped MWCNTs were formed using the CVD method. Next, the Fe_3_O_4_ nanoparticles (5–15 nm) were synthesized directly on the MWCNT surface in an MW-assisted process. The homogeneous distribution of nanoparticles on the surface of CNs, the formation of defects on the surface of MWCNTs during MW irradiation, and the doping of CNs with N improve the saturation magnetization of the resulting composites.

An MW irradiation was also used as an energy source to support the preparation of NiO [231], MnCo_2_O_4_ [204], and α-MoO_3_ [205,208] (Table 2). Nanoparticles of metallic oxides were directly grown on graphene sheets using the in situ MW irradiation method. A periodic repetition of MW irradiation through several cycles significantly increases the efficiency of the synthesis reaction, leading to large-scale processes [208]. The multi–step preparation of sulfides [197,203,212,213] may also be simplified by using MW–hydrothermal preparation of nanoparticles. In this case, it is not necessary to use chemical surfactants, and the preparation time is significantly reduced from several days to a few minutes. The homogenous distribution of CNs in solution was preceded by the partial exfoliation of graphene, which was carried out by MW irradiation. This approach ensured a homogeneous distribution of nanoparticles in the bulk of the composite. The nanoparticles were covered with graphene sheets or intercalated inside the materials. It, in turn, provides a conductive scaffold constructed from graphene sheets, increasing the material’s specific surface area and chemical stability. Hybrid composites’ porous and interconnected structures promote charge transport by encapsulating agglomerated metallic nanoparticles.

**Figure 12:**
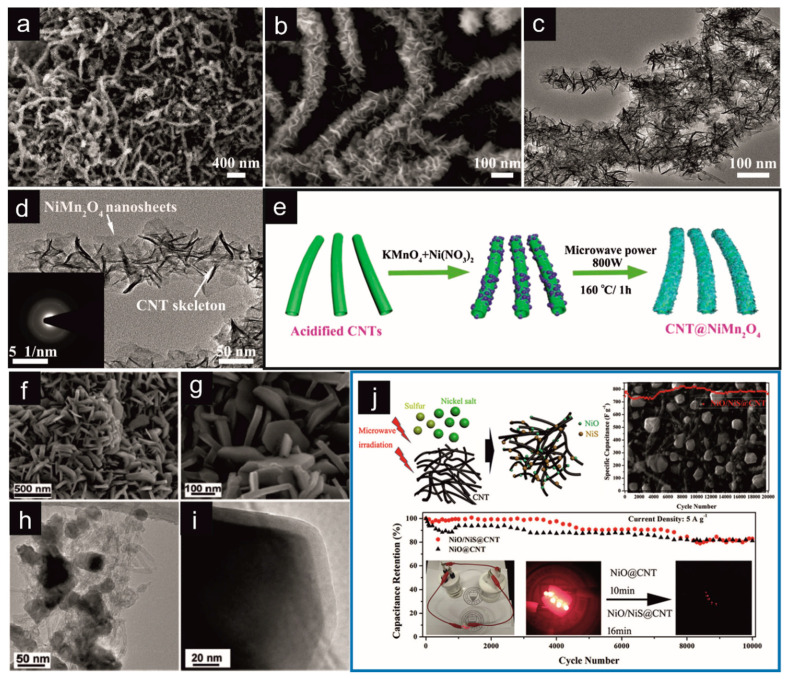
Schematic representation of the core-shell structures prepared by MW-assisted synthesis. (**a**,**b**) The low– and high–resolution FESEM images of CNT/NiMn_2_O_4_ composite (**c**,**d**) TEM images of CNT/NiMn_2_O_4_ composite; inset shows the selected area electron diffraction pattern. (**e**) Schematic representation of the formation of core–shell structures of the CNT/NiMn_2_O_4_ composite. Reprinted with the permission of Elsevier from Ref. [194]. (**f**,**g**) SEM and (**h**) TEM images of MWCNT/CoMoO_4_. (**i**) TEM image of individual CoMoO_4_ crystallite (core–shell structure). Reprinted with the permission from RSC, Ref. [195]. (**j**) Schematic illustration of the formation of NiS@CNT/NiO nanocomposites. SEM image of NiS@CNT/NiO. Reprinted with the permission from Elsevier, Ref. [196].

Using MW irradiation, it is also possible to obtain core-shell structures quickly [194,195,196]. In this case, the CNTs are surrounded by an inorganic phase, as shown in Figure 12a–d [194]. Briefly, in the first step, the oxidation of the MWCNTs was performed to increase their dispersibility and to introduce the nucleation sites for the inorganic nanoparticle creation. Next, a mixture of inorganic salt and an oxidizing agent was subjected to MW irradiation at elevated temperatures. Consequently, the walls of the MWCNTs were uniformly covered with an inorganic crystalline phase of various forms of crystallites. Depending on the irradiation power, temperature, or reaction time, the degree of coverage of MWCNTs varied. It is also possible to obtain a core–shell structure in the inorganic phase alone (Figure 12f–i). Then, two phases with different crystal structures are distinguished [195]. CoMoO_4_ nanopellets have a core-shell form with a well–crystallized bulk. The shell of CoMoO_4_ crystallite is amorphous. In both cases, the oxidized CNTs were utilized as MW absorbers and heterogeneous nucleation sites for inorganic nanocrystal formation. The MWCNT/CoMoO_4_ composite used as an electrode in the capacitors shows a *C_S_* of 170 F/g and a high cycling stability of 93.2% after 1000 cycles (Table 2) [195].

**Figure 13:**
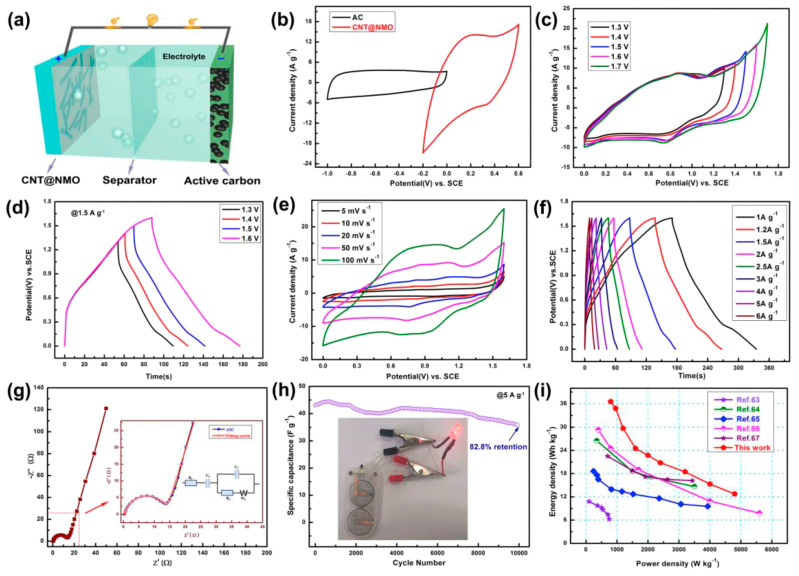
(**a**) Schematic diagram of CNT/NiMn_2_O_4_ asymmetric supercapacitor device. (**b**) Comparative CV curves of AC CNT/NiMn_2_O_4_ electrodes in a three-electrode system, 50 mV/s. (**c**) CVs of the device were measured at different potential windows, 50 mV/s. (**d**) The charge/discharge curves of the device were measured at different potential windows at 1.5 A/g. (**e**) CVs at the different scan rates from 0 to 1.6 V. (**f**) The charge/discharge curves measured at different current densities. (**g**) Electrochemical impedance spectra of the device. (**h**) Cyclic performance of the device at 5 A/g for 10,000 cycles. (**i**) The Regone plots of the CNT/NiMn_2_O_4_ and comparison with literature data. Reprinted with the permission from Elsevier, Ref. [194].

The CNT/NiMn_2_O_4_ composite synthesized by an MW–assisted hydrothermal process exhibited excellent electrochemical performance [194]. The electrochemical studies performed for these materials are shown in Figure 13. The CNT/NiMn_2_O_4_ electrode exhibits a high *C_S_* of up to 915.6 F/g at 1 A/g and an excellent cycling stability of 93.0% after 5000 cycles at 5 A/g. These properties are affected by the weaker crystallinity of NiMn_2_O_4_ and more defects and vacancies in CNTs. The CV curves exhibit similar electrochemical characteristics in the potential window from 1.3 to 1.6 V (Figure 13c). Increasing the voltage to 1.7 V and the OER and HER cause a jump in the charge current. It should be noted that the shape of the charge–discharge curves shows good linearity and symmetry in the potential range of 1.3–1.6 V (1.5 A/g), indicating the reversibility of the processes (Figure 13d). An asymmetric SC device with a positive electrode composed from CNT/NiMn_2_O_4_ shows the maximum energy density of 36.5 Wh/kg at *P* of 800 W/kg and cycling stability of 82.8% after 10,000 cycles at 5 A/g (Figure 13i). These parameters are higher than for other SC devices based on NiMn.

A multi-component nanocomposite consisting of CNTs, NiO, and NiS (NiS@CNT/NiO) was prepared quickly (60 s) using a one–step MW–assisted method (please see Table 2, Figure 12j) [196]. The different mass ratios of used substrates and MW conditions performed in the reaction were tested for their influence on the electrochemical properties of the created material. The composites containing both inorganic components, NiS, and NiO, showed optimal electrochemical performance. The NiS@CNT/NiO electrodes showed a *C_S_* of 809.7 F/g at 1 A/g and a cycling stability of approximately 100% retention after 20,000 cycles at 5 A/g. In the following example, MW–assisted hydrothermal synthesis was used to prepare the composite containing NiS and rGO (rGO/NiS) [197]. The MW–assisted synthesis eliminates chemical surfactants and reduces the preparation time from several days to only 6 h [197]. The electrodes fabricated from rGO/NiS exhibit an ultrahigh *C_S_* of 1745.7 F/g at 1 A/g and a high–capacity retention after 3000 cycles with high reproducibility.

The symmetric solid–state SC shows a *C_S_* of 14.20 F/g, an *E* of 7.1 Wh/kg and a *P* of 1836 W/kg (Table 2). The SC device based on N–rGO/NiS exhibits similar electrochemical performance with a *C_S_* of 1467.8 F/g at 1 A/g [203]. An asymmetric configuration of the capacitor, where N–rGO//N–rGO/NiS and graphite sheet as the current collector was used, characterizes the following parameters: a cyclic stability of 86.6% after 5000 cycles and an *E* of 66.6 Wh/kg and a *P* of 405.83 W/kg. Due to these parameters, the hybrid materials show potential for energy-storage applications.

The 3D S–rGO/NiFeS_2_ composite was synthesized using a one–step MW alcohothermal method, in which mercaptoacetic acid is used as a sulfur source for the doping of graphene and as a precursor of nickel–iron sulfide (Table 2) [210]. The composite shows a very porous structure and a large SSA, and thanks to chemical bonds with sulfur, it increases the binding of the nickel–iron precursor to the carbon material. These phenomena ensure the *C_S_* value of 1073.2 F/g at 1 A/g in 6.0 M KOH electrolyte. The asymmetric SC device, operating in the range of 1.6 V, delivers. The hybrid materials containing CN and Ni components show potential for energy–storage applications.

Simonkolleite (Zn_5_(OH)_8_Cl_2_·H_2_O) nanoplatelets were deposited on nickel foam–supported graphene (NiF–G/SimonK) using an MW–assisted hydrothermal method (Table 2) [212]. In this condition, the porous NiF–G/SimonK composite was obtained, in which the structural and morphological characteristic significantly affects the electrochemical performance. The NiF–G/SimonK composite, used as electrodes, exhibits a *C_S_* of 836 F/g at a *j* of 1 A/g and a cycling stability with capacitance retention of 92% after 5000 charge/discharge cycles. An MW–assisted synthesis was used to prepare a honeycomb–like graphene/NiCoS (G/NiCoS) composite [213]. The defect–rich structures of G/NiCoS ensure excellent electrochemical properties of this composite. The device with the electrodes made of G/NiCoS exhibits a high *C_S_* of 1186 F/g at 1 A/g and a capacity of 89.2% after 10,000 cycles, and an *E* of 46.4 Wh/kg. Due to their high energy and power density and excellent cycling stability, the multi–component hybrid materials show potential for energy–storage applications and electrocatalysis.

## 5. Concluding Remarks

The electrochemical properties of carbon–based electrode materials are closely related to their chemical composition, structure, and defects. They are ideal for catalysis, electrocatalysis, and electrochemistry due to their excellent conductivity and mechanical stability, sizeable specific area with micro– and mesoporosity, easy production, and high charged carrier mobility. They are also good candidates for electrocatalysis due to the easy formation of the defects in their networks that affect decreasing the reaction kinetic barriers while simultaneously increasing the efficiency and selectivity of the chemical transformation involved. From a chemical point of view, the essence of catalysis is breaking and re–forming chemical bonds under the influence of an external force. The ability to create carbon–carbon or carbon–heteroatom bonds in a simple way allows the use of this property, for example, in the production of fuels (H_2_ and O_2_).

Since all these parameters are easily achievable during the creation or modification of CNs, including during the irradiation of MW substrates, it is becoming increasingly used in this area. It was determined that not only the simplicity, reaction time, and the possibility of conducting it both in solution and the solid phase led to this, but above all the possibility of combining substrates of different chemical natures. Despite the many advantages of carbon-based materials, in some cases, it is necessary to combine them, for example, with an inorganic component, to achieve optimal final parameters. Also, in this respect, MW irradiation is an excellent source of energy that activates the chemical reaction between the organic and inorganic phases. The obtained materials have a homogeneous form because, as mentioned earlier, MW-assisted synthesis can be carried out in the solid phase, where the problem of solubility of organic and inorganic compounds in a given solvent is eliminated. Finally, the possibility of various combinations of synthesis parameters and substrates makes MW-assisted synthesis a universal method.

The review indicated that it is possible to create multi–component materials thanks to MW–assisted synthesis. Not only is it easy and quick to combine inorganic and carbonaceous materials, but often, the resulting materials exhibit unique structures and morphologies that need to be more attainable using conventional methods. Their physical and chemical properties make using them in many scientific fields possible. Here, we focused mainly on the electrochemical and electrocatalytic properties of the obtained materials because, in these areas, new solutions are constantly being sought to produce materials with optimal parameters. Because current scientific research indicates that in electrochemical and electrocatalytic processes of carbon–based materials, not only the surface morphology has a significant impact on the effectiveness of these reactions but also the heterogeneity of the structure; therefore, MW–assisted synthesis is considered one of the most promising methods in this area. Unfortunately, many of the solutions presented in this review still need to meet the parameters desired on an industrial scale.

When we think about industrial applications, we think about more than just the macro scale, which is often difficult to achieve, e.g., due to the kinetics of chemical reactions in large volumes. In this regard, we also need to take other factors into account. These certainly include the ease of producing materials, i.e., the simplicity of the technological process, the removal of intermediate products from the reaction mixture, limitations in using organic solvents, and low energy inputs in the entire technological process. All these parameters shape the product’s final price, i.e., in our case, a material with specific electrochemical and electrocatalytic properties. MW–assisted synthesis meets all the above assumptions and enables simple and cheap chemical reactions to achieve the intended synthesis goal. The examples of creating hybrid materials using MW irradiation presented here have great potential, and this trend should be developed in the future. However, the literature data presented are still within the scope of basic research, indicating that the transition from the micro- to macroscale is still a milestone.

## Data Availability

3rd Party Data.
